# Laparoscopic Management of Giant Ovarian Cysts Using the Alexis Laparoscopic System®: A Case Series

**DOI:** 10.3389/fsurg.2020.00024

**Published:** 2020-05-06

**Authors:** Jean Dubuisson, Sidney Heersche, Patrick Petignat, Manuela Undurraga

**Affiliations:** Gynaecological Surgery Unit, Department of Women-Children-Teenagers, University Hospitals of Geneva, Geneva, Switzerland

**Keywords:** Giant, ovarian cyst, laparoscopy, mini-invasive surgery, cap

## Abstract

**Objective:** The aim of this study was to review the characteristics of patients who underwent laparoscopic removal of giant ovarian cysts using the Alexis Laparoscopic System® and confirm the safety and feasibility of this technique.

**Method:** We conducted a retrospective review of data of women undergoing the procedure from March 2014 to February 2019. Inclusion criteria were ovarian cysts of at least 15 cm. Exclusion criteria were the presence of solid components and suspicion of neoplasia on imaging.

**Results:** Six patients were included in the series. Median size of the cysts at imaging was 22.8 cm (range 15–30 cm), while median volume was 5.9 L (range 1.9–15.6 L). Mean age of operated women was 59 years (range 21–88 years). All patients underwent exclusive laparoscopic management except one patient who underwent a conversion into midline laparotomy. The size of the skin incision initially performed to puncture the cyst ranged from 2.5 to 4 cm. On final pathological reports, two cysts were mucinous cystadenomas, and four were serous cystadenomas. There was no epithelial ovarian cancer or borderline tumor in any of the specimen operated.

**Conclusion:** Laparoscopic management of giant ovarian cysts using the Alexis Laparoscopic System® is safe and feasible in well-selected cases. Midline laparotomy can thus be avoided, decreasing the risk of post-operative complications and increasing quality of life of patients.

## Introduction

Benign giant ovarian cysts over 15 cm are rarely encountered nowadays because most are diagnosed and treated at an early stage. Standard surgical management requires a midline laparotomy to minimize the risk of cell spillage in case of unexpected malignancy. This surgical technique, though oncologically safe, is associated with an increase in morbidity, especially post-operative pain, as well as an increase in hospital length of stay. Laparoscopy is the treatment of choice in most benign ovarian cysts, but cyst size can be a limiting factor. A large ovarian cyst increases the complexity of a minimally invasive approach because of the difficulty in creating a pneumoperitoneum as well as a decrease in visibility and surgical mobility. Both of these factors can increase the risk of intraoperative spillage because of unintentional cystic rupture. Some case reports have been described in the literature with different surgical techniques to limit possible intra-abdominal spillage, but none of these procedures are suitable for giant cysts occupying the entire abdominal cavity (1–6).

Here we present a consecutive case series of patients with presumed benign giant ovarian cysts who underwent laparoscopic management using the Alexis Laparoscopic System® (Applied Medical, Rancho Santa Margarita, California, USA). We previously described this minimally invasive approach, with its potential of reducing morbidity while allowing oncologic safety (7).

The aim of this study is to review the mini-invasive technique using the Alexis Laparoscopic System® to remove the cysts and confirm its safety and feasibility.

## Method

This is a prospective follow-up of all patients presenting with a large presumed benign ovarian cyst operated by minimally invasive approach using the Alexis Laparoscopic System®. The studied period runs from March 2014 to March 2019. Written signed consent was obtained from all patients before surgery. Ethic Committee approval was not required because of the descriptive nature of the study. Pre-operative selection criteria were very strict. This approach was proposed to women presenting with large unilocular or multilocular ovarian cysts, larger than 15 cm, without solid components, showing no sign of malignancy at imaging (MRI or CT-scan). The size of the cyst was determined by MRI or CT-scan (larger diameter). In all cases, cysts reached the sub-costal area, excluding the possibility of creating a pneumoperitoneum through Palmer's point or other area in the upper abdomen. Thus, standard laparoscopy was considered as hazardous for these patients due to the risk of accidental cyst puncture or organ injury. Blood tumor markers (CA 125, CA 19.9 and CEA) were measured for each patient. Data was collected with specific emphasis on the size of the main initial incision, operative time, intra- and post-operative complications, conversion to laparotomy and the length of postoperative stay. The volume of the intracystic liquid drained intraoperatively was measured using the suction graduation. The size of the skin incision used to drain the cyst was measured at the end of the procedure with a graduated rule. Operative time was recorded from the cutaneous incision till total skin closure. Postoperative follow-up was performed at 10 days, 1 and 6 months. Postoperative complications related to the surgery were reported at 30 days.

**Figure 1:**
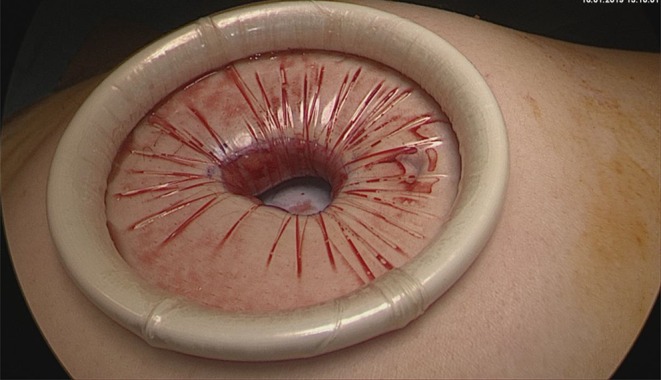
Umbilical open-laparoscopy to access the abdominal cavity. Visualization of the ovarian cyst wall. Insertion of the Alexis® retractor-protector.

**Figure 2:**
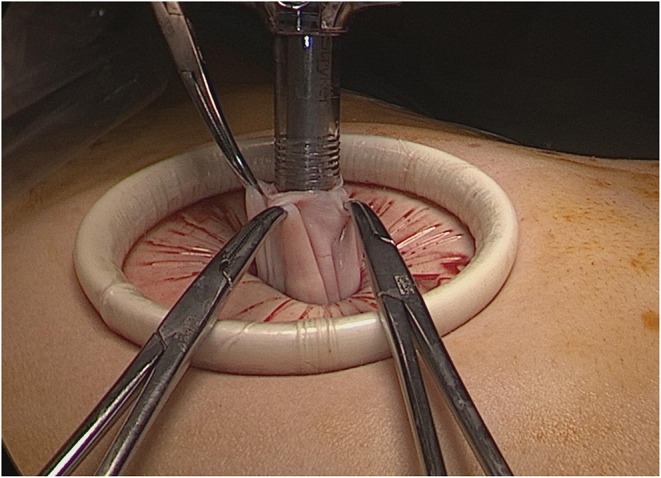
Contained cyst puncture under direct vision using a suction irrigation apparatus through a 12 mm trocar.

**Figure 3:**
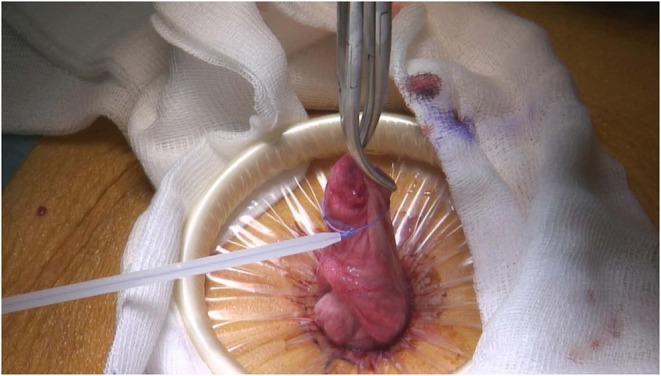
Closure of the puncture site using an Endoloop® ligature (Ethicon, Cincinnati, Ohio, USA).

**Figure 4:**
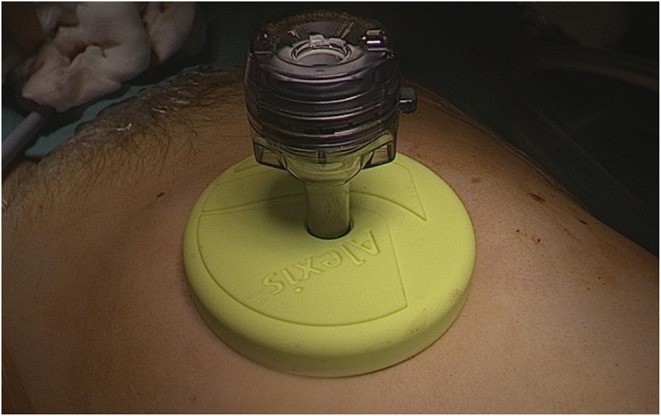
Setup of the Alexis Laparoscopic system® with a cap before creation of the pneumoperitoneum.

**Figure 5:**
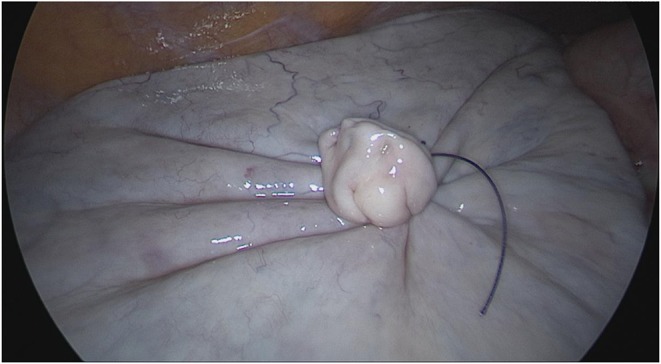
Laparoscopic intraabdominal visualization of the drained ovarian cyst.

The surgical procedure consists of performing a small supra umbilical or umbilical open-laparoscopy to access the abdominal cavity. The Alexis® retractor-protector (size S) is then inserted in the abdomen, and the cyst punctured with a trocar under direct vision (Figures 1, 2). After evacuation of all the intracystic liquid, a tight closure of the puncture site on the cyst is performed (Figure 3). A laparoscopic “cap” is then put on to create and maintain pneumoperitoneum and perform a standard multiport operative laparoscopy (Figures 4, 5).

## Results

Six patients were included in the series. Data are shown in Table 1. Mean age of operated patients was 59 years, including 2 pre-menopausal patients. Mean BMI was 22.1 (range 18.4–27). Three women were Physical Status score 3 according to the American Society of Anesthesiologists (ASA), one ASA 2 and two were ASA 1. Symptoms at presentation were varied. Most patients presented an increased abdominal girth and symptoms due to compression such as hydronephrosis, pollakiuria and intestinal sub-occlusion. Patient 6 was asymptomatic, while patient 2 presented with dyspnea.

**Table 1:** Patient's data.

**Patient**	**Age**	**Cyst size (cm)**	**Post-op. stay (d)**	**Incision diameter (cm)**	**Operative time (min)**	**Conversion to laparotomy**	**Histology**	**Post-op. complications**
1	84	30	8	3	190	Yes	Serous cystadenoma	No
2	63	21	4	3	152	No	Serous cystadenoma	No
3	67	21	12	4	193	No	Serous cystadenoma	No
4	88	23	6	3	160	No	Mucinous cystadenoma	No
5	33	25	1	3	60	No	Mucinous cystadenoma	No
6	21	17	1	2.5	80	No	Serous cystadenoma	No
Mean	**59**	**22.8**	**5.3**	**3**	**139**	**-**	**-**	**-**

All patients were diagnosed by imaging, either by MRI or CT-Scans. Median size of the cyst at imaging was 22.8 cm (range 15–30 cm), while median volume was 5.9 L (range 1.9–15.6 L). Four of the cysts were uniloculated, while 2 were multiloculated. None presented characteristics that could be suspect for neoplasia such as intracavitary vegetations, diffusion in restriction or solid portions. Five of the six patients presented with a normal CA 125 (average 24 kU/l, range 11–33). One patient presented with an elevated CA 125 of 146 (patient 3). Said patient also had a hydronephrosis as well as a sub-ileus because of the mass that could explain the elevated marker. Despite the elevated maker, a mini-invasive approach was chosen because she refused any primary invasive surgery by laparotomy. Three patients also had CA 19-9 and CEA values, all normal (patients 3, 4 and 6).

The size of the supra-umbilical or umbilical incision initially performed to drain the cyst ranged from 2.5 to 4 cm. Two patients underwent bilateral adnexectomy, three patients unilateral adnexectomy, and one patient an ovarian cystectomy. Mean operative time was 139 min (range 60–190 min). Average blood loss was 58 cc (range 0–200 cc). No cyst spillage occurred during the procedure. All patients underwent exclusive laparoscopic management except one patient (patient 3). A conversion to a 7 cm midline laparotomy was decided because of severe intraabdominal adhesions. Median post-operative stay was 5 days (range 1–12 days). Three patients (patients 1, 3 and 4) had significant longer post-operative stays due to their comorbidities and cognitive disorders, which explain the increased length of hospital stay. No patient presented any post-operative complications related to the surgery. On final pathological reports, two cysts were mucinous cystadenomas, and four were serous cystadenomas. There was no epithelial ovarian cancer or borderline tumor in any of the ovarian cysts operated.

## Discussion

As described by Dubuisson et al., the use of the Alexis Laparoscopic System® for the mini-invasive surgical extraction of large ovarian cysts is an alternative to the standard midline laparotomy in selected patients (7). Our results confirm the lack of specific per-and post-operative complications and the quick recovery period. The main incision size is substantially smaller than that of a laparotomy or a mini-laparotomy, and offers enough exposure to safely puncture the cysts in order to drain them without spillage. All masses were then extracted without having to increase the size of the incision. However, the main benefit of this technique is that it offers protection of the abdominal wound and it reduces the risk of cell spillage by using the wound protector-retractor. The conversion rate was low, concerning only one patient with severe intraabdominal adhesions.

Few, heterogeneous reports have been documented in the literature describing similar techniques. Only 5 of these series included cysts of at least 15 cm, as in our series (1–6). Some of these case series included a larger number of patients, but at the same time, they also included malignant cysts in up to 13% of cases, as described by Ki et al. In our opinion, we cannot stress enough the need for strict selection criteria to decrease the risk of an accidental finding of invasive tumor in the final pathological specimen. The validation of this technique is necessary through multicentric studies, in order to increase the number of patients analyzed.

## Conclusion

Laparoscopic management of giant ovarian cyst using the Alexis Laparoscopic System® is a safe and effective procedure, and should include only very well-selected patients presenting with benign preoperative criteria. In our experience, the presence of multiples intracystic locules is not a limiting factor for this mini-invasive procedure while severe intraabdominal adhesions appears to be the main limit conducting to conversion to laparotomy.

## Data Availability Statement

The datasets generated for this study are available on request to the corresponding author.

## Author Contributions

JD: conceived and designed the analysis, collected the data, and wrote the paper. SH: collected the data and wrote the paper. PP: contributed data and designed the analysis. MU: collected the data, performed the analysis, and wrote the paper.

## Conflict of Interest

The authors declare that the research was conducted in the absence of any commercial or financial relationships that could be construed as a potential conflict of interest.
